# Dental Education

**Published:** 1889-06

**Authors:** Eugene H. Smith

**Affiliations:** Boston


### International Dental Journal.

Vol. X. June, 1889. No. 6.

### Original Communications.[1]

### DENTAL EDUCATION.[2]

BY EUGENE H. SMITH, M.D., D.M.D., BOSTON.

No one comes in contact with candidates for graduation in dentistry who is not compelled to admit that there is need for a more thorough training for students before admission into practice. The deficiency is only too apparent, but the remedy is not so plain. Decidedly different views are held as to the required additions to our present accepted curriculum in order to bring about the desired result. Among dental practitioners we find two extremes: the first relies entirely upon mechanical means, the second upon theory for the basis of practice. Both these positions are to be deprecated, and it is a question as to which class has done the most harm. The mechanical practitioner, wholly devoid of any knowledge of the physiological principles that underlie his work, has destroyed hundreds of teeth, and brought about the consequent evils. The dental medical practitioner, with mechanical ideas not only foreign, but considered quite beneath him as a professional gentleman, has placed fillings in the teeth he would save, as he would drop pills into the stomach of his patient, or has dosed them with some nostrum that he was quite sure was the antidote for further decay, and contemplated the great possibilities of the profession when all its practitioners are doctors of medicine, whilst his failure to save

[1] The editor and publishers are not responsible for the views of authors of papers published in this department, nor for any claim to novelty, or otherwise, that may be made by them. No papers will be received for this department that have appeared in any other journal published in this country. The journal is issued promptly on the 15th of the month.

[2] Read before the American Academy of Dental Science, Boston, Mass.

teeth is quite as apparent as that of his mechanical brother. Dentistry as I see it, requires to a great degree a practical training, and the successful man must possess in the beginning manipulative ability of a high order. For certain it is—however much some may wish to deny it—mechanical skill is largely at the foundation of our calling.

To raise this calling to a higher plane is the duty of us all, and I think it is with this end in view that many good men have obtained for themselves, and are strongly advocating for others, the degree of medicine; while some, I regret to say, have obtained it simply and solely as a means supposed to elevate them above their fellows. This is no idle assertion, for I assure you as a fact that I have been addressed by men considered quite willing to obtain it, even at a slight mental cost, on the ground of its giving the holder a higher professional atmosphere and admission into the ranks of physicians. I assert, without fear of well-founded contradiction, that our best practitioners of to-day are quite as likely to be found outside of those holding the medical degree, and that the better practitioners now holding that degree are not indebted to it for their superiority as dentists. American dentistry is indebted almost wholly for its rapid advancement in the past seventy years and its recognition by the public as an important calling to the practical ingenuity of the leading men, and its continued advancement, in my opinion, must come through the same channel.

Our methods of treating irregularities are far from perfect, and must be developed, if developed at all, through mechanical means; for beyond the physiological principles underlying the movement of the teeth, I cannot see that medical knowledge can be of any great use. It is so in the filling of teeth and in the large majority of the operations now performed, or which are advisable to be performed, by the dentist. The mistake which I think the advocates for the medical degree for dentists make is, in assuming that the holding of it elevates the standing of the profession. It elevates it only in that it merges it with another profession that already takes a high standing among the learned professions; but the high standing in this case was brought about through those men that were in the beginning liberally educated—men possessing a collegiate education. The ministry was considered for a long time the most learned calling, and why? Simply because it had among its devotees more bachelors of arts than any of its sister professions. This is now changed, or changing, and learned men are found in great numbers in other callings. Our own profession needs the influence of this class of men, and when they come, as come sometime they will, then the practical work will lose some of its present odium—odium attached to it, through the false ideas of antiquated thinkers—then will our profession rank among those considered learned.

Every teacher in our dental schools will agree, I think, with me that where students fail the most is in the practical portion of the work. To-day it is no uncommon thing to find students that pass all the theoretical branches with great credit, and yet have the utmost difficulty in passing the practical departments, and no teacher of dentistry, however strongly he may think the degree of medicine necessary, can conscientiously pass a poor manipulator. This fact alone proves that more time is actually needed in the practical departments, and all observers of the present results of dental education, I think, will concede it. To accomplish this more time must be added for work in the practical departments. A course of three full years is really necessary for training in the manipulative part of dentistry and in the studies pertaining strictly to dental practice. In order to obtain a liberal education as advocated by those who hold for the possession of a medical training, besides the degrees in arts and dentistry, requires at least ten years' study. Is dentistry, I would ask, a profession of such magnitude as to require more training for its practice than any of the other professions? I think not—consequently we must trim the curriculum advocated.

The question then arises, what can we best give up? A liberal education is at the foundation of everything scientific and learned, consequently this cannot be cut out, and as I have already affirmed that the dental studies require more, rather then less time, then there is no chance for trimming in that direction, but I do see a chance in the medical department. For I cannot see any practical use in the dental student's studying obstetrics, diseases of women, skin diseases, the minute anatomy of the body, nor entering into the more complex problems of physiology and other medical studies that I might add to this list; but the medical advocate replies, if you throw them out you forfeit the medical degree. I admit it; I am willing that it should be forfeited. If the degree is of no practical use for us as dental practitioners, why should we wish it? I think the notion of a medical education for a dental student found its place in the minds of men through the fact that a medical education was obtained by many of our early dentists; this was greatly to their credit, for at that time dental schools were unknown, and they got what theoretical knowledge they could of dentistry through medicine—certainly it was that in pursuing this course they got but very little knowledge of our specialty; and they themselves found it necessary to establish special schools for more thorough training; and from that time through their influence we have made most wonderful advancement, and continued progress must come, so it seems to me, through the perfecting of these special schools.

Some of the graduates in medicine have said they hoped to see the time when the degree of medicine would be required of every dental candidate. Some of these same gentlemen could have put their time to a better advantage than getting the medical degree by making up for some of their deficiencies in the more common branches of study. I should much rather see the time when our dental schools could increase their curriculum, and require the degree of A.M. Many States, as you know, require of the practitioner a degree in medicine or in dentistry, before he is allowed to practice dentistry. This is excellent in one way, and all wrong in another. The idea that a doctor of medicine, without any dental training is fitted to practice dentistry, is preposterous; yet, all these laws are so framed as to admit it. Let a student enter one of our medical colleges with no knowledge of dental practitioners' being in existence, and no outside means of ascertaining the fact during his course of study, we all know he could graduate, and still be entirely ignorant of our profession. In my own practice I have had cases where physicians have treated neuralgia of different parts of the body for weeks and months, when the whole cause of the trouble was to be found in some dental lesion.

A case recently came into my hands where a physician had been treating with internal exhibition of medicine, an abscess of an upper incisor, for malaria; and although the patient showed no other sign of this disease he pronounced it local malaria at that point, and so dosed her accordingly. It is needless to say that the local malaria, so-called, readily yielded to the proper dental treatment. Many other cases, equally absurd, I could mention, which have come under my observation. Now, the many cases of the kind that are on record show conclusively, that an education in medicine simply by no means fits a man for the practice of dentistry, and that the clause in the laws of States admitting men so educated to the dental practice should be repealed.

In closing, allow me briefly to express my ideas of the elevation of dentistry. First, a preparatory education, which must be liberal, and which, in my opinion, can best be obtained in an institution where, together with a liberal education, manual training is made an important branch; for, as I have said before, I believe mechanical skill is really at the foundation of dentistry, and this skill must be born with the child, or else acquired very early in life. From here to the special dental school, where enough of the medical science is taught so that the dentist will learn sufficient to know when a trouble of the mouth belongs to the surgeon proper, and turn it over accordingly; where special dental structures are more thoroughly taught, and where his previously acquired mechanical skill can receive that special training needed to make a skilled operator and a fine mechanic. When this time comes, you will see the elevation of your profession called, learned with the others, and with this advantage: that our science will be the most exact.

